# Behavioral Manipulation for Pest Control

**DOI:** 10.3390/insects12040287

**Published:** 2021-03-26

**Authors:** Valerio Mazzoni, Gianfranco Anfora

**Affiliations:** 1 Fondazione Edmund Mach, Research and Innovation Centre, 38010 San Michele all’Adige, TN, Italy; 2 Center for Agriculture, Food, Environment (C3A), University of Trento, 38100 San Michele all’Adige, TN, Italy

Pest control is moving towards a dramatic reduction in pesticide-based approaches in favor of more eco-friendly strategies characterized by the promotion of ecological intensification of agriculture and reduction of human inputs (especially pesticides) [1,2]. Behavioral manipulation is perfectly suitable to assist in these tasks, since it is based on communication disruption techniques aimed at interfering with the common habits of the principal pests in order to minimize their negative impacts on crop production [3]. Nowadays, the use of semiochemicals (i.e., pheromones and allelochemicals) is a consolidated practice, used worldwide on many different crop systems; on the other hand, semiophysicals (i.e., substrate-borne vibrational signals) are emerging as a new technology, which is quickly gaining considerable interest in both producers and industries with business in crop protection [4].

Behavioral manipulation for pest control involves the use of natural and/or artificial signals, such as pheromones, kairomones, sounds and vibrations, to interfere with fundamental behaviors, such as feeding and mating [5,6]. These techniques fit well with the concept of a multidisciplinary approach and allow a strong and synergic interaction between apparently distant disciplines such as biology, ecology, mechanics, chemistry and informatics. In this context, the current Special Issue will consider the following topics:

- **Kairomone-based lures to attract noxious insects into traps**.

Traps are no longer passive and time-consuming tools that must be checked periodically by specialized personnel; rather, they are being replaced by smart-tech devices. Integrated cameras and sensors now deliver considerable amounts of data in real time, 24 h a day. Thanks to camera lenses, which can achieve high sensitivity, even minuscule species such as thrips can be feasible targets [7].

The development of new and improved blends is a crucial aspect through which to maximize trap efficiency. In particular, bioactive volatiles, produced by bacteria that work as bio-catalyzers, are powerful synergists to kairomone-based baits. An example of this is the lactic acid bacterium, *Oenococcus oeni*, which significantly improves the attractiveness of commercial food baits of the Spotted Wing Drosophila, *Drosophila suzukii* [8,9].

- **Pheromone traps to estimate pest population density**.

The lack of exact correspondence between the number of captured individuals and the actual population size of a pest has long been an important limitation in the use of pheromone traps in monitoring programs within IPM strategies. Mathematic models can provide a solution to this issue by estimating the probability of a localized infestation through the number of trap captures, as in the case of the Gypsy Moth, *Lymantria dispar*. By designing suitable monitoring grids, it would be possible to fill this gap and to provide crucial information for decision-making [10].

- **Vibrational signals as a tool for mating disruption**.

The playback of rivalry signals is a perfect example of a species-specific approach to pest control with a null impact on the environment. Insects such as stinkbugs, which communicate at medium and short range with vibrational signals, rely on the perception of co-specific substrate-borne signals to identify and locate a potential partner. By introducing disruptive signals, which mimic natural stinkbug female rivalry signals, into the host plants, it is possible to affect the pair formation process and thus to prevent mating [11].

The playback of specific and generic signals (i.e., white noise, music) can also affect the mating behavior of insects. In the case of the potato psylla, *Bactericera cockerelli*, a vector of zebra chip disease, the playback of conspecific female calls to the host plant significantly decreased mating success, whereas the use of unspecific signals affected mate finding [12].
